# Larval growth of the polychaete *Arenicola marina* under different temperature and food conditions: consequences on bioenergetic models

**DOI:** 10.1093/conphys/coac033

**Published:** 2022-06-09

**Authors:** Coralie Broquard, Théo Lancelot, Sébastien Lefebvre, Lucie Courcot, Sylvie M Gaudron

**Affiliations:** 1 UMR 8187 Laboratoire d’Océanologie et de Géosciences (LOG), Université de Lille, ULCO, CNRS, IRD, F-59000 Lille, France; 2 Sorbonne Université, UFR 927, F-75005 Paris, France

**Keywords:** lugworm, growth rate, larval stages, DEB model, biphasic model, Arrhenius temperatures

## Abstract

*Arenicola marina*, a marine benthic polychaete, is widespread on sandy beaches in Europe and considered as an ecosystem engineer despite commonly used as bait by fishermen. Data regarding the bioenergetics of the lugworm larval stages are still incomplete. Trochophore is initially lecithotroph and then becomes planktotroph while growing as metatrochophore on subtidal area, a quite stable daily temperature environment compared with the foreshore, where juveniles and adult live, with daily temperature fluctuating up to 15°C. These discrepancies in temperature ranges may influence the temperature corrections (TCs) that control metabolic rates during the life cycle of *A. marina*. We carried out laboratory experiments in microcosms by inducing artificial spawning of lugworms, and then undertaken *in vitro* fertilization to obtain embryos and, finally, to follow, the larval development up to 10 segments with chaetae for 50 days under three temperature conditions (13°C, 15°C and 17°C) and two food conditions (‘fed’ and ‘non-fed’). The first feeding (‘birth’) of *A. marina* larvae was deciphered anatomically for a size between 450 and 500 μm and described at 17 days post-fertilization for larvae reared at 15°C and 17°C. Using a biphasic model with a von Bertalanffy growth before ‘birth’ and an exponential growth after ‘birth’, among the three temperature treatments, the 15°C condition exhibited the best larval performance. TC based on embryonic and larval metabolic rates gave an Arrhenius temperature of ~6661 K and a higher boundary temperature tolerance range of ~294.5 K. Both temperature values differ from those calculated from TC based mostly on juvenile and adult metabolic rates. We claim to use two sets of Arrhenius temperatures according to the life history stages of *A. marina* while using Dynamic Energy Budget model. This model was developed initially in order to manage the conservation of the lugworm species.

## Teaser

Biphasic growth models of *Arenicola marina* larvae highlight an optimal temperature at 15°C with a lower temperature tolerance range compared with juveniles and adults. We claim that two sets of Arrhenius temperatures depending on the life history stages should be implemented when using an abj-Dynamic Energy Budget model in this species.

## Introduction

Polychaeta are mainly marine metazoans and represent significant part of the benthic biomass (Grémare *et al.*, 1998). They play a major role in the functioning of benthic ecosystems and serve as bio-indicators of the marine environment health status (Giangrande *et al.*, 2005; Sivadas *et al.*, 2010). Polychaetes have a market value in fisheries where they are used as bait by fishermen (Watson *et al.*, 2017). In aquaculture, polychaetes may be used either as food supplements due to their high nutritional value for cultured aquatic species (Pairohakul *et al.*, 2021) or by their abilities in waste depollution in integrated aquaculture (Jansen *et al.*, 2019; Jerónimo *et al.*, 2020). Finally, studies have shown the therapeutic interest that certain species of polychaetes may have for applications in human health (Kuijk and van Die, 2010; Singh *et al.*, 2014). For all these reasons, the breeding and marketing of polychaetes are of growing interest and are current issues (Micael *et al.*, 2016; Olive, 1993; Olive, 1994). This attractiveness causes intensive harvesting of these species, mainly on foreshore, which results in an alteration of the environment and therefore has a deleterious effect on benthic ecosystems (Beukema, 1989; Clarke *et al.*, 2017). Moreover, this overexploitation of the resource endangers the survival of some species of polychaetes (Cole *et al.*, 2018; De Cubber *et al.*, 2018). To overcome these issues, some countries have implemented regulations that aimed at regulating the rate of withdrawals over the years in sensitive areas, e.g. in Portugal (Xenarios *et al.*, 2018), USA (Sypitkowski *et al.*, 2009), Australia (Cole *et al.*, 2018) and UK (Watson *et al.*, 2015). Another way to avoiding the depopulation of polychaetes is to develop the domestication of species of high economic interest; thus, farms of *Atilla virens* (Olive, 1999; Sustainable Feeds Ltd™, 2018), *Arenicola marina* (Olive *et al.*, 2001), *Arenicola defodiens* (Olive *et al.*, 2001), *Hediste diversicolor* (Bischoff *et al.*, 2009), *Diopatra aciculata* (Safarik *et al.*, 2006), *Perinereis cf. nuntia* (Poltana *et al.*, 2007) and *Perinereis helleri* (Palmer *et al.*, 2016) have emerged. However, a complete knowledge of the physiology of these polychaetes and in particular of the early stages of their development is necessary to carry out these conservation and cultivation projects.

The lugworm *A. marina* (Linnaeus, 1758) is one of the most used bait for professional and recreational fishing in Western Europe, where it is intensively harvested from the Arctic to the Mediterranean (De Cubber *et al.*, 2018; Watson *et al.*, 2017). Moreover, the strong affinity of its haemoglobin for oxygen has led to the production of this worm for therapeutic uses in human health, whether as an organ preservative during transplants but also as a possible blood substitute (Batool *et al.*, 2021; Rousselot *et al.*, 2006). *Arenicola marina* lives in 5–40-cm deep U-shaped burrows in soft foreshore sediments in the intertidal area, from mediolittoral to infralittoral (De Cubber *et al.*, 2020). The life cycle of *A. marina* has been described in details (De Cubber *et al.*, 2019; Farke and Berghuis, 1979a, 1979b; Newell, 1948; Newell, 1949; Reise *et al.*, 2001). Juveniles and adults live in burrows, where they swallow the sediment at the surface being psammivorous. Lugworms may move backwards in the burrow, where they expulse their faeces by their pygidium that forms a characteristic sand-pile called castings. Breeding season occurs in autumn where lugworm’s population have annual epidemic spawning of few days (Watson *et al.*, 2000). Females spawn their oocytes within the gallery, while males release sperm puddles on to the sediment surface that will be diluted by the incoming tide and then drawn into female’s gallery by pumping. Fertilization takes place inside the gallery (Williams *et al.*, 1997) where embryos remain until hatching at the trochophore larval stage. Trochophores and then metatrochophores are lecithotrophic larvae dispersing several days (depending on temperature) in the water column until temporally (few months) settling on subtidal marine habitats such as macroalgae or mussel beds (De Cubber *et al.*, 2019; Farke and Berghuis, 1979a, 1979b). During that first settling period, the first food intake [‘birth’; Dynamic Energy Budget (DEB) theory see after; Kooijman, 2010] occurs, where larvae will live in a mucus tube but going out of their tube to collect organic matter or phytoplankton. Larvae will develop segments with chaetae called setigers (up to 19 setigers) until the completion of metamorphosis that could last up to 7 months (De Cubber *et al.*, 2019; Farke and Berghuis, 1979a, 1979b). When metamorphosis will be completed, a second phase of dispersal will occur into the water column allowing post-larval stages to reach the foreshore. These post-larvae will then settle on high part of the shore, burrowing themselves and becoming a psammivorous juvenile as the adults. While growing and acquiring maturity to become an adult, lugworms will migrate lower on the shore (De Cubber *et al.*, 2020). Although the overall functioning of the life cycle is known, knowledge was still poor regarding the fine tune of the larval stage development of *A. marina* on the subtidal area (De Cubber *et al.*, 2019; Farke and Berghuis, 1979a, 1979b; Newell, 1948).

To overcome this, and thus have a better knowledge of the different life stages of this species, an abj-DEB model was developed by De Cubber *et al.* (2019). Indeed, DEB models allow to predict the physiological processes (such as growth, assimilation, respiration, reproduction) of a species across its whole life cycle according to environmental conditions (such as food availability and temperature) (Kooijman, 2010). When applying DEB theory (Kooijman, 2010), abj-DEB model (Marques *et al.*, 2018) differs from a standard DEB model by adding an extra juvenile life stage that takes place between the first feeding of the larval stage (birth, ‘*b*’) to the end of the metamorphosis (‘*j*’) at the post-larval stage, where metabolic acceleration (*s_M_*) occurs leading to an exponential growth of the individual (Kooijman, 2014), compared with a classical von Bertalanffy growth before ‘birth’ and from the juvenile to adult stages (Kooijman, 2010). However, data used for the abj-DEB model developed by De Cubber *et al.* (2019) were not supported by data for the early life stages between the trochophore and the post-larval stages despite some predictions of age and length were obtained by simulation. No experimental studies have described so far, the early larval stages of *A. marina* into details regarding the age versus length according to temperature and food level. Most studies were focused on fertilization success and temperature effect during embryogenesis (prior the trochophore stage) at a stage that embryos still live into the female gallery on the foreshore (Lewis *et al.*, 2002; Watson *et al.*, 1998). In addition, abiotic factors such as temperature and food availability have not been tested in the laboratory to determine their effect on larval growth and development.

Thus, we carried out an experimental study in laboratory in order to deepen our knowledge on the influence of temperature and food on the physiology of the larval stages of *A. marina*. The study aimed to determine precisely when the first feeding (‘the birth’: in DEB theory) occurs (age at ‘birth’ and length at ‘birth’) in order to describe the biphasic growth before and after ‘birth’ according to different temperature and food conditions. The second goal of this paper was to decipher if there was a difference into the thermal tolerance during the life cycle of *A. marina* between different life stages as larval stages live in the subtidal areas, a quite stable daily temperature, whereas juveniles and adults live in the intertidal areas, where daily temperature can fluctuate up to 15°C. These discrepancies in temperature ranges in these two marine habitats may result in different sets of Arrhenius temperatures (Kooijman, 2010) that control metabolic rates of the lugworms according to its life stage. Overall data could be used to improve the existing abj-DEB model that has been developed initially in order to help stakeholders to make decision for preserving *A. marina* in areas with high anthropogenic pressure or to improve the farming of this species in aquaculture.

## Materials and methods

### Study area and sampling

For the need of our experiment, 180 adult lugworms were collected at Wimereux (50°46′N, 1°36′E), located on the Eastern English Channel, part of a marine-protected area (MPA) created in 2012. The coastline is principally composed of sandy beaches as well as rocky shores mainly colonized by algae and mussels on the intertidal and subtidal areas (Rolet *et al.*, 2015). In this MPA, adult population of *A. marina* is found on the high and mid-shore (De Cubber *et al.*, 2018). From 2 to 16 September 2019, 180 adults of *A. marina* were sampled in total, using a shovel and a bait pump (Decathlon ltd.) on the mid-shore at low tide.

### Broodstock selection and maintenance

At the Wimereux Marine Station, collected lugworms (*n* = 180) were maintained in a 300-l tank with a continuous seawater flow (300 l.h^−1^), placed on a thermostatically controlled room (15°C). A continuous flow of water mixed the tank for 24 hours in order to clean the worms by removing sand and micro-algae residues. Then, to assess the reproductive status of each worm, biopsies of the coelomic fluid were performed using a sterile hypodermic syringe on anaesthetized individuals *A. marina* in three successive ethanol solution (1%, 2.5% and 5%) in twice-filtered seawater solutions (TFSW, 0.45 μm and 0.2 μm) (Gaudron and Bentley, 2002). Observations using an optic microscope (Motic® BA210) allowed to establish the state of maturity of the gametes and to differentiate the sexes. After sex determination, males and females were separated and kept in two different tanks with continuous seawater flow. While maintaining the lugworms, regular gametes observations using the optic microscope were carried out randomly on biopsies of five males and five females in order to estimate the reproductive status of each individual. For females, 30 random oocytes were measured using the optic microscope equipped with Motic Image Plus© 3.0 software. Female gametes were estimated to be ready for fertilization when mean oocytes diameter was at 180 μm (Watson *et al.*, 1998). For male gametes, maturity was fixed when 80% rate of spermatocytes was in the morula stage (Dillon and Howie, 1997).

### Spawning induction, artificial fertilization

Five females and five males with the most mature gametes were selected as broodstock for artificial fertilization. Lugworms were washed with autoclaved TFSW and then placed in individual tanks (15.0 × 8.0 × 10.0 cm) filled with 1 l of TFSW. Each selected female was injected with two prostomial homogenates (Howie, 1961) and kept for 24–48 hours in an individual tank at 15°C until the release of the oocytes. After spawning, females were removed from their tanks and oocytes were collected with a 63-μm mesh. Then, female gametes were washed twice with TFSW and stored in 5 ml microtubes at 4°C.

Just after the release of oocytes, each male was injected with two prostomial homogenate (Pacey and Bentley, 1992) and monitored until gametes release. After ejection by male’s nephridiopores, ‘dry’ sperm was collected immediately with a micropipette and placed in 1 ml microtubes on ice (Williams *et al.*, 1997). Male gametes were counted using a Neubauer counting chamber (Sigma ltd.) under the optic microscope. Before the artificial fertilization, female (*n* = 5) and male (*n* = 5) gametes were pooled together to increase fertilization success. Approximately 10^6^ oocytes were mixed with a concentration of 10^4^ sperm per egg in a 2-l autoclaved glass container filled with 1 l of TFSW for a 10-minute sperm–egg contact time to avoid polyspermy (Williams *et al.*, 1997). Then, fertilized oocytes were removed and washed twice with TFSW before being distributed (~10^5^ oocytes per container) in 10 different 1-l autoclaved glass containers filled with 500 ml of TFSW and placed in the dark at 15°C.

### Experimental design for larval rearing

After 48 h post-fertilization, TFSW was changed every two days with embryos retained and washed in a 63-μm mesh. Some subsamples were fixed in 4% formaldehyde for further observations. From Day 4, the larvae began to secrete a lot of mucus, and to avoid clogging, they were gently resuspended with Pasteur pipette every day until Day 12.

On Day 6, the 10 glass containers (1 l filled with 500 ml of TFSW) were placed in three different thermostatically controlled rooms with respectively 3 glass containers at 13°C and 17°C and 4 glass containers at 15°C.

After 24 hours of acclimation of these new temperature conditions at Day 7, one glass container per room at 13°C and 17°C and two glass containers at 15°C were supplemented with a solution of microalgae (4.10^4^ cell/ml concentration of RGcomplete APBreed™, Planktovie ltd.) every 2 days and called the ‘fed’ conditions, while the remained containers at 13°C, 15°C and 17°C were called the ‘non-fed’ conditions. TFSW was changed initially every two days but after Day 22 it was extended to 3–5 days. The experiments lasted for 50 days.

### Monitoring of larval morphology and biometry

The larval development from artificially fertilized oocytes was monitored daily for the first three weeks, then twice a week thereafter, using the optic microscope equipped with Motic Image Plus© 3.0 software. Times required reaching the following stages of trochophore and metatrochophore were recorded for each temperature (13°C, 15°C and 17°C) and food conditions (‘fed’ and ‘non-fed’). For each temperature condition, 15–30 larvae per glass container were collected at random and sacrificed for morphological observations and biometry. The selected larvae were anesthetized (Gaudron and Bentley, 2002). The observation of the number of setigers (segments bearing setae), as well as the opening of the mouth, the anus and the appearance of the digestive tract were carried out using the optic microscope. In addition, taking photographs allowed to measure the total length of each larva (Motic Image Plus© 3.0 software).

Scanning electron microscope (SEM) was used for better visualization of ontogeny. For this, some larvae fixed in 4% formaldehyde were washed in MilliQ water (Millipore) in 40-μm mesh and were gradually dehydrated by placing them successively for 1 hour in ethanol (Merck, Normapur) baths ranging from 30% to 100% with a step of 10%. Following this dehydration, and in order to fix and dry the larvae, they were put twice in a row, for one hour, in a bath of hexamethyldisilazane (HMDS, Molekula). The larvae were collected individually using micro forceps and stuck on aluminium stubs (Agar Scientific) with double sticky carbon tabs (Agar Scientific), which was finally sputter coated under Argon flow with Au/Pd (Polaron SC 7620) for 90 seconds. SEM observations were carried out under the SEM LEO 438 VP using a secondary electron detector for topography at 20 keV.

### Definition of ‘birth’

The date of the first exogenous food intake, called ‘birth’ (DEB theory; Kooijman, 2010) in our study, corresponds to the concomitant appearance of the opening of the mouth, of the anus and the appearance of the gut. Initially, *A. marina* larvae are lecithotroph living on maternal reserve and this is called the ‘embryo’ stage in the DEB theory (Kooijman, 2010) having a von Bertalanffy growth curve. Then when the larva starts to feed on exogenous food (planktotroph) by developing a functional gut, the growth is exponential until the end of the metamorphosis. The transition between a lecithotrophic larva and the feeding larval stage has been described for each temperature conditions through microscopic observation.

### Biphasic bioenergetic modelling

Larval growth was modelled using a biphasic time-dependent model described by a set of two equations. The change depends on the time of ‘birth’ (*tb*), where growth before ‘birth’ follows the laws of von Bertalanffy (von Bertalanffy, 1957) (Equation 1) and, after ‘birth’ it is exponential (Equation 2).

For length data, the growth equation is written as follows:

(1) \begin{align*} {L}_1(t)&={L}_{inf}-\left({L}_{inf}-{L}_0\right)\ast ex{p}^{- bt}\ \textrm{with}\nonumber\\ {L}_{inf}&=\frac{a}{b}\ \textrm{for}\ t\le tb\ \textrm{i.e.{before}}\ {\text{`
birth'}} \end{align*}

(2) \begin{equation*} {L}_2(t)={L}_1(tb)\ast ex{p}^{ct}\ \textrm{for}\ t > tb \ \textrm{i.e. after `birth'} \end{equation*}

*L_1_(t)* and *L_2_(t)* are lengths as a function of time (*t*) and *L_0_* and *L_1_(tb)* are length at time 0 and at *tb,* respectively. ${L}_{inf}$ is the asymptotic length, *a* and *b* are the size-specific rates of energy acquisition and energy use for body maintenance (the von Bertalanffy growth rate between fertilization and *tb*), respectively, and *c* the exponential growth rate after *tb*.

### Temperature range for metabolic responses in larval stages

All metabolic rates depend on body temperature (Kooijman, 2010), and in ectotherms it corresponds to the external temperature such as in polychaetes. Thus, a temperature correction (TC) is usually applied on metabolic rates using the Equation (3), where *T_A_* is the Arrhenius temperature (in K), *T_ref_*, the reference temperature (293.15K), and *T* is the experimental temperature (in K):

(3) \begin{align*} \mathrm{TC}=\exp \left(\frac{T_A}{T_{ref}}-\frac{T_A}{T}\right) \end{align*}

**Figure 1:**
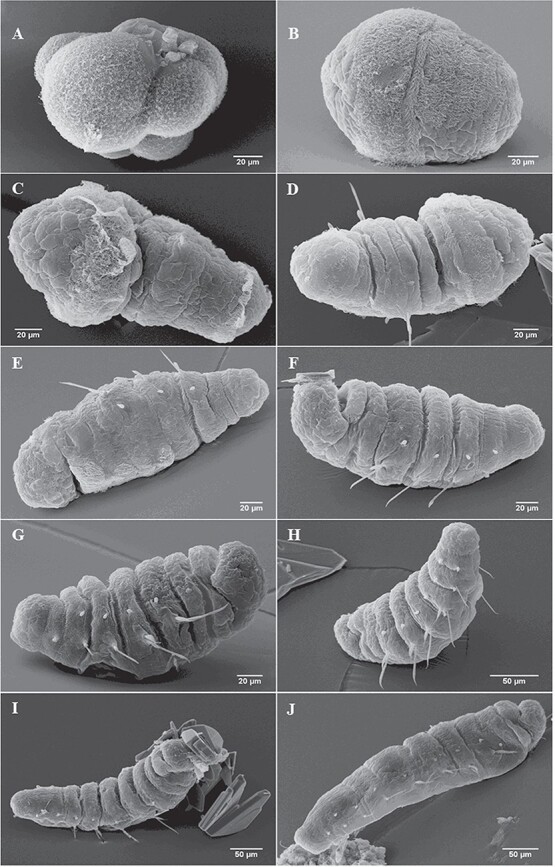
Scanning electron microscopy photographs of 10 larval stages of *A. marina*. (A) Embryo at early stage of cell division before hatching; (B) trochophore stage after hatching; (C) metatrochophore with 1 setiger (segment with chaetae); (D) 2 setigers; (E) 3 setigers; (F) 4 setigers; (G) 5 setigers; (H) 6 setigers; (I) 7 setigers; (J) 8 setigers.

**Table 1:** Larval development of *A. marina* at 13°C, 15°C and 17°C. At 7 dpf (Time), larvae were fed with microalgae. Larval stages correspond to the number of setigers (S). Total length is the mean of the n replicates with its standard deviation (±).

Temperature	Time (days)	Larval stage	Total length (μm)	*n*	Mouth and anus opening	Presence of a digestive tract
15°C	0–2	Embryo	159 ± 8	73	No	No
	3	Trochophore	169 ± 14	91	No	No
	6	1S	255 ± 28	48	No	No
	9	2S	356 ± 15	8	No	No
	12	3S	383 ± 34	12	No	No
	14	4S	476 ± 33	23	Yes	No
	17	4S	465 ± 52	22	Yes	Yes
	20	5S	506 ± 66	16	Yes	Yes
	24	6S	499 ± 86	16	Yes	Yes
	43	7S	541 ± 157	6	Yes	Yes
	50	8S	746 ± 258	22	Yes	Yes
13°C	8	2S	246 ± 11	5	No	No
	13	3S	415± 30	10	No	No
	16	4S	471 ± 69	8	Yes	No
	21	5S	510 ± 46	5	Yes	Yes
	24	6S	461	1	Yes	Yes
	43	7S	674 ± 76	4	Yes	Yes
	50	7S	800 ± 129	5	Yes	Yes
17°C	8	2S	276 ± 19	11	No	No
	11	3S	371 ± 21	9	No	No
	14	4S	454 ± 30	19	Yes	No
	16	5S	445 ± 40	8	Yes	No
	17	5S	459 ± 45	10	Yes	Yes
	21	6S	588 ± 95	6	Yes	Yes
	37	7S	551 ± 155	18	Yes	Yes
	43	8S	586 ± 204	20	Yes	Yes
	50	10S	544 ± 186	21	Yes	Yes

Outside the lower and higher boundaries of the species-specific temperature tolerance range (respectively *T_L_* and *T_H_*), the TC shape differs and is calculated adding an extra term to the Equation (3) as presented in Equation (4), with *T_AL_* the Arrhenius temperature below the lower boundary of the species-specific temperature tolerance range (in K) and *T_AH_* the Arrhenius temperature above the higher boundary of the species-specific temperature tolerance range (in K) (Kooijman, 2010).

(4) $$ \small{\begin{align*} {\displaystyle \begin{array}{l}\mathrm{TC}=\\ {}\exp \left(\frac{T_A}{T_{ref}}-\frac{T_A}{T}\right)\left[\raisebox{1ex}{$1+\exp \left(\frac{T_{AL}}{T_{ref}}-\frac{T_{AL}}{T_L}\right) +\exp \left(\frac{T_{AH}}{T_H}-\frac{T_{AH}}{T_{ref}}\right)$}\right.\\\left.\!\left/ \!\raisebox{-1ex}{$1+\exp \left(\frac{T_{AL}}{T}-\frac{T_{AL}}{T_L}\right)+\exp \left(\frac{T_{AH}}{T_H}-\frac{T_{AH}}{T_{ref}}\right)$}\right.\right]\end{array}} \end{align*}}$$

A simpler version of this equation for the higher boundary- of the temperature tolerance range only is as follows:

(5) $$ \small{\begin{equation*} \mathrm{TC}=\exp \left(\frac{T_A}{T_{ref}}-\frac{T_A}{T}\right)\ast \left[\raisebox{1ex}{$1+\exp \left(\frac{T_{AH}}{T_H}-\frac{T_{AH}}{T_{ref}}\right)$}\!\left/ \!\raisebox{-1ex}{$1+\exp \left(\frac{T_{AH}}{T_H}-\frac{T_{AH}}{T_{ref}}\right)$}\right.\right] \end{equation*}}$$

The Arrhenius temperature of *A. marina* has been previously estimated, using Equation 3, together with other DEB parameters using the DEBtool package (De Cubber *et al.*, 2019; Marques *et al.*, 2018). In addition, the temperature tolerance range and the Arrhenius temperatures of Equation 4 have been estimated for the species from data collected mainly in juveniles and adults (De Cubber *et al.*, 2020). Hence, new data on larval stages of *A. marina* were used to re-estimate the Arrhenius temperatures, i.e. *T_A,_ T_AH_* and to estimate the higher boundary of the temperature tolerance range, *T_H_*, using Equation 5. As no data were available below 5°C, it was not possible to estimate the lower boundary of Equation 4. The new data set consisted in the parameters (*a*, *b* and *c*; Equations 1 and 2) of the biphasic growth model at 13°C, 15°C and 17°C as well as the data from several fertilization success experiments carried out at 5°C, 10°C, 13°C, 15°C, 18°C, 20°C and 22°C by Lewis *et al.* (2002). Each data set was standardized by its maximum value to get values between 0 and 1 in line with the TC (Equation 5).

### Statistics and fittings

All growth curve fitting processes and associated statistics were coded in R version 4.0.3 (2020). A nonlinear least squares method (package ‘nls2’; Grothendieck, 2013) was used to fit Equations 2 and 5 as it allows multiple starting values to avoid local minima problems in parameter estimation. This package provides parameter best estimates and standard errors, and parameter significances by *t*-test. Further, bioenergetics models were tested for either differences in the temperature effect (3 modalities) or differences in the food condition (‘fed’ and ‘non-fed’) within each temperature (2 modalities) following the method of Ritz and Streibig (2008) and using analysis of variance (ANOVA). For the temperature factor, the sum of the residual sum of squares (RSS_ind_) of the three fitted models for each temperature (three parameters per model, nine in total, ‘n_par_ind_’) were compared with the RSS_all_ of a model grouping all data and fitted with only 3 parameters (‘n_par_all_’). For the food condition factor, we assumed there was no effect of food condition before *tb* and then, the von Bertalanffy phase of the biphasic model has *L_inf_* and *b* as common parameters for a given temperature. Hence, the sum of RSS_ind_ of the two fitted models for each food condition (two common parameters plus one *c* parameter per model, ‘n_par_ind_’ = 4) were compared with the RSS_all_ of a model grouping all data for a given temperature and fitted with only three parameters (‘n_par_all_’ = 3).

The *F* statistic was calculated as follows:

$$ F=\frac{\frac{RSS_{all}-{RSS}_{ind}}{\left(N-n\_{par}_{all}\right)-\left(N-n\_{par}_{ind}\right)}}{\frac{RSS_{ind}}{N-n\_{par}_{ind}}} $$

With *N* denotes the total number of individuals. The *P-*value was then determined by searching for the *F-*value in the *F* distribution with degrees of freedom ($n\_{par}_{ind}-n\_{par}_{all},N-n\_{par}_{ind}$) using the function ‘pf’ of the R statistical package.

## Results

### Effect of temperature on larval development of *A. marina*

According to the 13°C, 15°C and 17°C exposed temperature respectively, the chronology of *A. marina* larval development (Fig. 1) and their biometry were recorded (Table 1). The fertilized oocytes had an average diameter of 176 ± 6 μm and develop to embryo by cell division during embryogenesis (Fig. 1A). Larvae hatch at the trochophore stage (Fig. 1B) at the end of the gastrulation at 3 days post-fertilization (dpf) with a mean total length of 169 ± 14 μm (Table 1). The larvae developed their first setiger at 6 dpf (Fig. 1C) with a mean total length of 255 ± 28 μm, becoming a metatrochophore. All larvae were still conditioned at 15°C at that time (Table 1). After 6–50 dpf, the larvae were raised to three different temperature conditions (13°C, 15°C and 17°C), and the appearance of new setigers (up to 10 setigers) were not tuned between the three treatments. Indeed, at 15°C the larvae have developed 4 setigers (S) at 14 dpf and 6 S at 24 dpf (Table 1; Figs. 1F,H). While the larvae placed at 13°C needed 16 and 24 dpf to reach 4 S and 6 S, respectively (Table 1), those placed at 17°C reached 4 S and 6 S at 14 and 21 dpf, respectively (Table 1). At 50 dpf, larvae had reached 7 S (Fig. 1I) with a mean total length of 780 ± 130 μm (Table 1) at 13°C, 8 S (Fig. 1J) with a mean total length of 746 ± 258 μm (Table 1) at 15°C and 10 S with a mean total length of 544 ± 186 μm (Table 1) at 17°C. Although, there was a time lag of larval development as a function of temperature, larvae had equivalent size for each stage. Indeed, for example for the 4 S stage, larvae measured 471 ± 69 μm, 476 ± 33 μm and 454 μm ± 31 μm at 13°C, 15°C and 17°C, respectively (Table 1).

### Effect of diet on larval development of *A. marina*

The dietary transition between lecithotrophic larvae to planktotrophic larvae (‘birth’) occurred at a size between 450 and 500 μm, regardless of temperature (Fig. 1F; Table 1; first time that a row has ‘yes’ in the last two columns). In terms of duration, the age at ‘birth’ has taken place at 17 dpf for the larvae reared at both 15°C (4 S stage) and 17°C (5 S stage) and at 21 dpf for those placed at 13°C (5 S stage) (Table 1).

**Figure 2:**
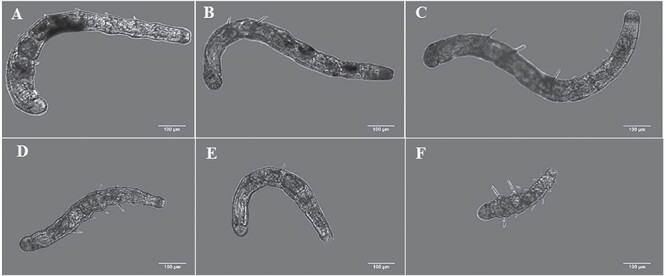
Images obtained with an optic microscope of *A. marina* larvae at different food levels and temperature conditions at 50 dpf. (A) 13°C and ‘fed’ conditions; (B) 15°C and ‘fed’ conditions; (C) 17°C and ‘fed’ conditions; (D) 13°C and ‘non-fed’ conditions; (E) 15°C and ‘non-fed’ conditions; (F) 17°C and ‘non-fed’ conditions.

Growth retardation was observed visually at 50 dpf between larvae fed with microalgae (‘fed’) and those non-feds whatever the temperature treatments (Fig. 2). For the three-temperature conditions, the mean total lengths of larvae at 50 dpf of the ‘non-fed’ conditions were lower than those of the ‘fed’ conditions (Table 2), but at 13°C mean total length (659 ± 96 μm) of the ‘non-fed’ condition, was not significantly different than that of the ‘fed’ condition (800 ± 129 μm) (*t*-test; *P* = 0.08); at 15°C, the mean total length of larvae from ‘non-fed’ condition (487 ± 110 μm) was highly significantly different than that of the ‘fed’ condition (746 ± 258 μm) (*t*-test; *P* < 0.001); at 17°C, the mean total length of larvae from ‘non-fed’ condition (506 ± 212 μm) was not significantly different than that of the ‘fed’ condition (544 ± 186 μm) (*t*-test; *P* = 0.56). It seems that some shrinkage had occurred in larvae from the ‘non-fed’ treatments between 43 dpf to 50 dpf both at 15°C and 17°C (Table 2).

**Table 2:** Larval development after ‘birth’ according to diet condition for each temperature treatment. Times correspond to days post-fertilization. Total length is the mean of the n replicates with its standard deviation (±).

Temperature	Time (dpf)	Total length (μm) for ‘non-fed’ condition	Total length (μm) for ‘fed’ condition
13°C	21	551 ± 74 (*n* = 12)	505 ± 51 (*n* = 4)
	24	543 ± 79 (*n* = 13)	461 ± na
	27	594 ± 65 (*n* = 8)	497 ± 64 (*n* = 9)
	29	626 ± 132 (*n* = 7)	463 ± 25 (*n* = 2)
	37	651 ± 120 (*n* = 14)	561 ± 64 (*n* = 5)
	43	626 ± 102 (*n* = 11)	674 ± 76 (*n* = 4)
	50	659 ± 96 (*n* = 12)	800 ± 129 (*n* = 5)
	17	465 ± 84 (*n* = 12)	465 ± 52 (*n* = 22)
	21	468 ± 80 (*n* = 7)	472 ± 34 (*n* = 7)
	24	465 ± 71 (*n* = 12)	499 ± 86 (*n* = 16)
	27	511 ± 95 (*n* = 19)	485 ± 78 (*n* = 13)
	29	514 ± 113 (*n* = 15)	467 ± 68 (*n* = 15)
	37	556 ± 77 (*n* = 13)	583 ± 85 (*n* = 14)
	43	613 ± 154 (*n* = 9)	541 ± 157 (*n* = 6)
	50	487 ± 110 (*n* = 23)	746 ± 258 (*n* = 22)
15°C 17°C	17	476 ± 57 (*n* = 11)	459 ± 45 (*n* = 10)
	21	500 ± 70 (*n* = 11)	588 ± 95 (*n* = 6)
	24	420 ± 70 (*n* = 10)	485 ± 66 (*n* = 8)
	27	466 ± 133 (*n* = 10)	505 ± 107 (*n* = 12)
	29	463 ± 72 (*n* = 11)	473 ± 57 (*n* = 8)
	37	514 ± 125 (*n* = 13)	551 ± 155 (*n* = 18)
	43	687 ± 265 (*n* = 16)	586 ± 204 (*n* = 20)
	50	506 ± 212 (*n* = 17)	544 ± 186 (*n* = 21)

**Figure 3:**
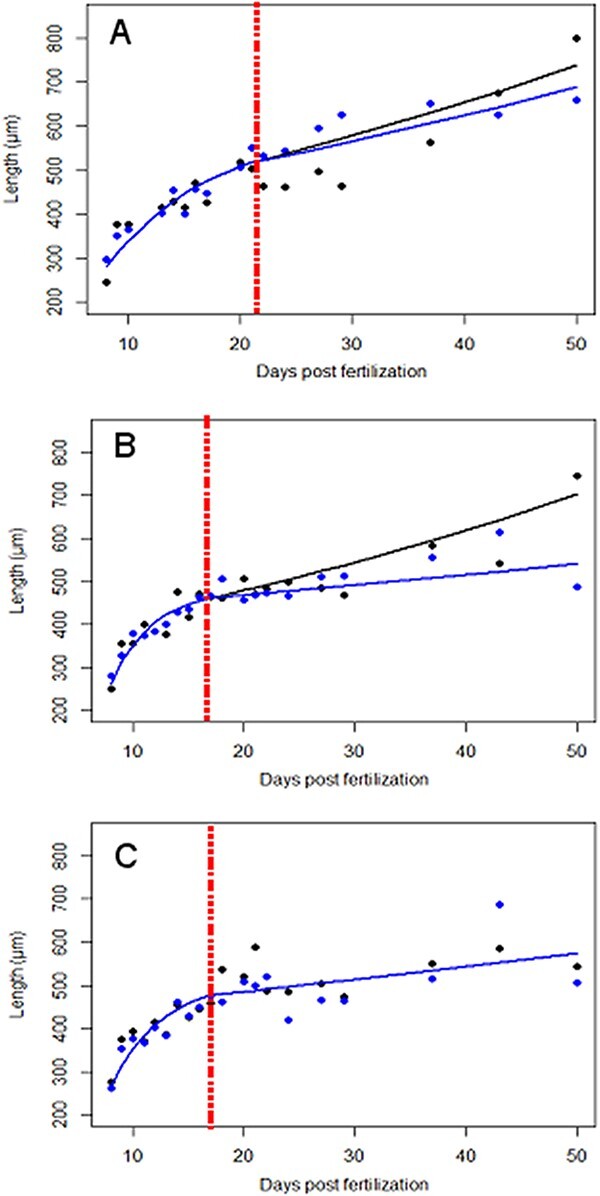
Evolution of the total length of *A. marina* larvae at three different temperatures following days post-fertilization. (A) At 13°C, where the age at first food intake (‘birth’) is indicated by the red dotted vertical line (21 days); (B) at 15°C, where the age at first food intake (‘birth’) is indicated by the red dotted vertical line (17 days); (C) at 17°C, where the age at first food intake (‘birth’) is indicated by the dotted red vertical line (17 days). Lines are simulations of the models: classic von Bertalanffy (first phase of the biphasic growth model) and exponential (second phase of the biphasic growth model). Larval growth for the ‘fed’ condition is in black and for the ‘non-fed’ condition is in blue.

### Effect of temperature and diet conditions on bioenergetic of *A. marina* larvae

Temperature had a significant effect on the biphasic growth models (*F*_(6, 1338)_ = 9.72; *P* < 0.001). In the first phase of the model (von Bertalanffy), growth rate *b* gave better performance at 15°C (0.263 d^−1^) and 17°C (0.216 d^−1^) compared with 13°C (0.107 d^−1^), whereas in the second phase of the model, exponential growth rates *c* were greater at 13°C (0.012 d^−1^) and 15°C (0.013 d^−1^) compared with 17°C (0.006 d^−1^) (Table 3; Fig. 3). The effect of food on the biphasic growth model is highly significant at 15°C (*F*_(1, 556)_ = 59.44; *P* < 0.001), where the growth model gave better results in ‘fed’ conditions compared with the ‘non-fed’ condition (Fig. 3B). The effect of food is marginally significant at 13°C (*F*_(1, 285)_ = 2.77; *P* = 0.097), but still the biphasic growth model gave better performance in ‘fed’ condition compared with the ‘non-fed’ condition (Fig. 3A). At 17°C, there is no effect of the food conditions on the biphasic growth model (*F*_(1, 494)_ = 0.0; *P* = 0.98), where both biphasic models were similar given bad performance regarding larval growth (Table 3; Fig. 3C).

**Table 3:** Biphasic growth modelling parameters where *a* and *b* are from von Bertalanffy’s phase and *c* results from the exponential phase. The parameter *a* was estimated from *L_inf_* and b using Equation 2.

Treatment	Parameters	Unit	Value	Standard error	*P-value*
13°C	*L_inf_*	μm	595.83	43.77	<0.001
	*a*	μm/d	63.71	-	-
	*b*	/d	0.107	0.026	<0.001
Fed	*c*	/d	0.012	0.001	<0.001
Non-fed	*c*	/d	0.010	0.001	<0.001
15°C	*L_inf_*	μm	481.59	15.02	< 0.001
	*a*	μm/d	126.92	-	-
	*b*	/d	0.263	0.051	<0.001
Fed	*c*	/d	0.013	0.001	<0.001
Non-fed	*c*	/d	0.005	0.001	<0.001
17°C	*L_inf_*	μm	512.61	29.15	<0.001
	*a*	μm/d	110.61	-	-
	*b*	/d	0.216	0.060	<0.001
Fed	*c*	/d	0.006	0.001	<0.001
Non-fed	*c*	/d	0.006	0.001	<0.001

### TC on metabolic rates of *A. marina* across different life history stages

**Figure 4:**
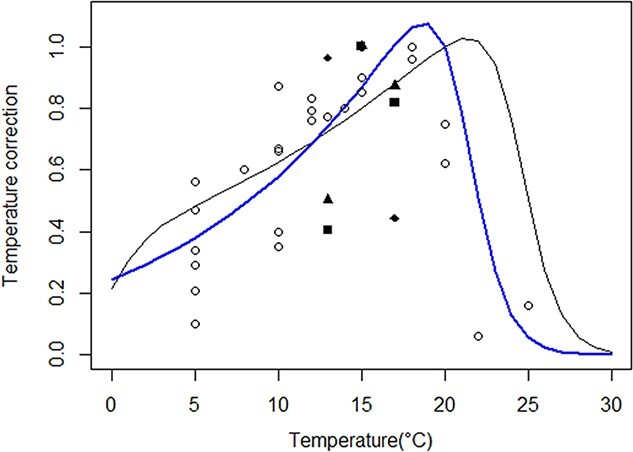
TCs in different life stages of *A. marina*. The black line represents the TC used in the abj-DEB model mostly on juvenile/adult stages from De Cubber *et al.* (2020) and the blue line represents the TC using datasets from fertilization success rate (circle; Lewis *et al.*, 2002) and from this study on larval growth with parameters from the first phase of the biphasic growth model [*a* = black triangle; *b* = black square; equation (1)]and from the second phase of the biphasic growth model [*c* = black diamond; equation (2)] at several temperatures.

The estimates of the biphasic larval growth models (Table 3) along with data from Lewis *et al.* (2002) after being standardized by their maximum values, helped to re-estimate the TCs using Equation 5. As *T_AH_* was non-significant in the first regression fit (*P* = 0.14), *T_AH_* (82 380 K) from De Cubber *et al.* (2020) was set in the Equation 5. New TCs were calculated with a new *T_A_* equaled to 6661.79 K (± 1241.5; *P* < 0.001) and a new *T_H_* equaled to 294.44 K (±0.42; *P* < 0.001). Overall, these new Arrhenius temperature datasets were different from those of De Cubber *et al.* (2020), where the *T_H_* from this study (blue line; Fig. 4) issued from larval metabolic rates, was lower than that of De Cubber *et al.* (2020) issued from juvenile/adult metabolic rates (black line; Fig. 4), and the *T_A_* from our datasets (slope of the blue line on the left part of the curve; Fig. 4) was higher than that of De Cubber *et al.* (2020) (slope of the dark line on the left part of the curve; Fig. 4).

## Discussion

The present work shows that a cohort of *A. marina* was successfully fertilized *in vitro* getting the embryo stage and then reared for 50 days under favourable experimental growth conditions allowing the cohort to hatch to different larval stages including both a lecithotrophic stage using maternal reserves and then, using the exogenous food provided by the algal culture being then planktotrophic. This experiment enabled to strengthen knowledge on the first life history stages of the lugworm species *A. marina* and, in particular, the precise age and length of the occurrence of the ‘birth’ stage with the biphasic growth before and after ‘birth’ under temperature and food control conditions. These data will be useful to consolidate the abj-DEB model developed by De Cubber *et al.* (2019, 2020).

### Early larval stages of *A. marina* and ‘birth’ stage

Most of earlier work on developmental larval stages of *A. marina* were reported (Newel, 1948, 1949; Farke and Berghuis, 1979a, 1979b) at a time that the species delimitation between *A. marina* and *A. defodiens*, a sympatric species that might occur at some beach in European marine habitats, was not yet known (Cadman and Nelson-Smith, 1993; De Cubber *et al.*, 2018) mixing the different ontogeny larval stages between the two species. The best study on larval development was carried out by Farke and Berghuis (1979b) in laboratory where authors develop a genius microsystem enabling mature adult lugworms (supposedly *A. marina*) to spawn and larvae to develop in the laboratory. However, timing of spawning events, larvae occurrence and control of temperature conditions could not be recorded precisely. Despite this, previous authors (Farke and Berghuis, 1979b) described nicely the behaviour, habitats and biometry of three larval stagesof *A. marina*. Newly hatched trochophorelarvae were in female gallery and had a size ~0.25 mm in length. In our study, the trochophore larval stage was lesser in length and it was the larvae of one setiger that reached 0.25 mm. Metatrochophores with 3S were seen swimming by ciliary movements and measured ~0.5 mm (Farke and Berghuis, 1979b). In our study the 3S larval stage was ~0.4 mm, close to what was measured by Newel (1948, 1949) from its *in situ* sampling larvae of *A. marina*. After this 3S stage, larvae started to secrete a mucus tube in order to adhere to hard substrate and they changed their behaviour (Farke and Berghuis, 1979b). Larvae could leave their mucus tube in order to crawl and feed on particles deposited around the tube being deposit-feeder (Farke and Berghuis, 1979b). Only larvae with 6S were shown to contain food particles in their gut with a size of 0.8 mm (Farke and Berghuis, 1979b). In our study the first food intake (‘the birth stage’) was observed earlier at the 4S/5S larval stage at a size between 450 and 500 μm. Marty *et al.* (1997) had followed the appearance of setigers following time in the larvae of the polychaete *H. diversicolor*. Larvae of 3S (425 ± 30 μm) were starting to feed (‘birth’) on non-fertilized oocytes in females gallery being cannibalistic and adelphophagic. This length at first feeding is very close to that observed in the larvae of *A. marina*.

Within an abj-DEB model, two primary parameters depend on the metabolic acceleration (*s_M_*) that occurs between the ‘birth’ stage to the end of the metamorphosis (Kooijman, 2010, 2014): (i) the maximum assimilation rate after metamorphosis ${\Big\{{\dot{p}}_{Am}\Big\}}_j={\Big\{{\dot{p}}_{Am}\Big\}}_b\ {s}_M$ and (ii) the energy conductance values ($\dot{v}$) after metamorphosis ${\dot{v}}_j={\dot{v}}_b{s}_M$. The metabolic acceleration is calculated as the ratio of the structural length at metamorphosis to the structural length at ‘birth’: ${s}_M={L}_j/{L}_b$. Within the abj-DEB model developed on *A. marina* (De Cubber *et al.*, 2019), the physical length at ‘birth’ (*Lw_b_*) was set at 230 μm (twice lower to what is observed in this study) and this might have changed the estimation of the metabolic acceleration ${s}_M$. In this study, we managed to describe precisely the length at ‘birth’ (~450 μm) and this will complete the dataset of the abj-DEB model of *A. marina* developed by De Cubber *et al.* (2019).

### Effect of abiotic factors on the first food intake (‘birth’) in *A. marina*

When the larvae hatch at the trochophore stage, and until the development of the complete digestive tract occurring at the 4S/5S metatrochophore stage, the larvae draw their energy from the yolk reserves (lecithotrophy) for growth, maintenance and the complexity of its maturity in DEB theory (Kooijman, 2010). Thus, the availability of food in the environment has no influence on the transition from the lecithotrophic stage to the planktotrophic stage (‘birth’), but temperature does. According to our results, ‘birth’ appeared earlier in metatrochophores subjected to warmer temperatures (17 days at both 15°C and 17°C) compared with lower temperature (21 days at 13°C). This is not in line with the age at ‘birth’ estimated by the abj-DEB model proposed by De Cubber *et al.* (2019), where simulation carried at 10°C gave a ‘first feeding’ at 10.52 dpf, twice much lower than that observed at 13°C. However, when De Cubber *et al.* (2019) simulated the temperature conditions for a whole year at Wimereux (Eastern English Channel) using real *in situ* data from 5.5°C to 20°C, a closer simulated value of the age at ‘birth’ was estimated (15.5 days closed to the 17 days observed for 15°C in our experimental set up). In the field, Newell (1948, 1949) observed metatrochophores of *Arenicola* sp. ready to become planktotrophic at 2–3 weeks post-spawning at Whistable (UK). In this study we managed to describe precisely the age at ‘birth’ for three different temperatures and this will complete again the dataset of the abj-DEB model of *A. marina* developed by De Cubber *et al.* (2019).

### Abiotic factors on growth rates of *A. marina* larvae

The increase in seawater temperature has induced an acceleration of larval development giving at 50 dpf, metatrochophores with more developed setigers (10S) in higher temperature conditions (17°C) compared with lower temperature, e.g. at 13°C only metatrochophores with 7 segments with chaetae were recovered. Thus, larvae reared at 17°C changed larval stages faster than those exposed at 13°C meaning the energy allocated to the complexity of the larvae was greater (*E_H_* in DEB theory; Kooijman, 2010). However, the mean total length of the larvae reared at 17°C (~544 μm) was lower compared with those reared at 13°C (~800 μm) at 50 dpf meaning in DEB interpretation that less energy was allocated to somatic growth while more energy was allocated to the complexity of the larvae reared in higher temperature. The discrepancy in mean length was enhanced by the poor food conditions treatment (‘non-fed’) that induced a kind of starvation at 50 dpf for both 15°C and 17°C treatments. In DEB theory (Kooijman, 2010), energy is needed in priority for maintenance of maturity and growth when less energy is available from mobilization; what is seen here is the larvae seem to shrink and some lysis of cells might have occurred.

In this study, the first phase (von Bertalanffy growth) of the biphasic growth model of *A. marina* larvae that encompasses trochophores and metatrochophores up to 3S (before ‘birth’) was better at 15°C and 17°C. These larval stages occur first within the female gallery on the intertidal foreshore and then disperse in the water column. Then, after ‘birth’, at the larval stage of 4S, the second phase (exponential growth) of the biphasic growth model was greater at 13°C and 15°C, where at these larval stages, *A. marina* larvae are living on the subtidal areas. For both biphasic growth phases, the optimal temperature was shown to be at 15°C before and after ‘birth’. Lewis *et al.* (2002) found for different populations of lugworms in the UK that the optimal temperature for fertilization success (embryos stages) was between 15°C and 18°C. Lewis *et al.* (2002) were quite astonished by their results as spawning periods of *A. marina* occurred at lower temperature in the UK (10–12°C), where embryos develop in female gallery on the intertidal habitat. Lewis *et al.* (2002) concluded that lugworms were not breeding at their optimal temperature and other selective pressures were certainly acting. In our study, the optimal temperature was found at 15°C and this, for others life history stages (trochophores and metatrochophores) of *A. marina* that live not anymore on the intertidal area but on the subtidal area (Farke and Berghuis, 1979a; Newell, 1948, 1949). At Wimereux (Eastern English Channel), *A. marina* population spawns from the end of September to early October (De Cubber *et al.*, 2018) where temperature drop from 15°C to 14°C but larvae seems to be in their optimal temperature at least during the onset of larval development as temperature fall in winter to temperatures up to 5.5°C (De Cubber *et al.*, 2019). At a regional scale, other populations of *A. marina* breed later until mid-November on the Eastern English Channel (De Cubber *et al.*, 2018). In mid-November, temperature is ~10°C as seen in the UK in Lewis *et al.* (2002). *Arenicola marina* populations are widespread in Europe and some population live in South of Europe such as in Portugal (Pires *et al.*, 2015) where the mean seawater temperature is much higher in winter but in the range of the optimal temperature for larvae and in spring and summer in the range of juvenile/adult optimal temperature. This may explain the well establishment of this species in South of Europe, where in Portugal the lugworm is seen as an invasive species (Pires *et al.*, 2015).

### Applications in DEB theory and in aquaculture

Intertidal species (mostly ectotherms) such as polychaetes, bivalves and gastropods can experience during low tide a great variation (up to 20°C) of daily temperature either in winter or in summer (De Cubber *et al.*, 2020; Moisez *et al.*, 2020; Seuront *et al.*, 2019) compared with species living in a more stable daily temperature environment such as in the subtidal area. As reported by Kooijman (2010), these species have enzymes involved in metabolic reaction that function in this broad temperature range with the consequence to have a relatively low Arrhenius temperature (*T_A_*) (~6000 K), compared with species that live in more constant daily temperature having a higher Arrhenius temperature (~12 000 K). *T_A_* calculated using DEB tool (Add-my-pet-database) of the polychaete *H. diversicolor* and the cockle bivalve *Cerastoderma edule*, living both on intertidal mud flat, were found respectively to be 4877 K and 5290 K. De Cubber *et al.* (2020) have estimated a *T_A_* of 4014 K for *A. marina*, a correct value for an intertidal species. The calculation was based on metabolic rates of life history stages of the lugworms (embryos, juveniles and adults) that live on the foreshore. In our study, a new set of Arrhenius temperatures (*T_A_* and *T_H_*) was calculated based on TCs of metabolic rates of only early-life stages of *A. marina* (embryos and larvae). *T_A_* of early life stages of *A. marina* that spend most of their time in the subtidal area (a more stable environment), as expected, was found higher (~6661 K) compared with the *T_A_* (~4014 K) (De Cubber *et al.*, 2020) of life stages of *A. marina* that live on the foreshore (a highly variable environment). Likewise, the higher boundary temperature value (*T_H_* = 294.4 k; ~21.25°C) of the early life stages was lower than that of the juvenile/adult stages (*T_H_* = 297.7 k; ~24.55°C; De Cubber *et al.*, 2020). As already reported by Kooijman (2010), larvae of intertidal species that live in pelagic environment have a higher Arrhenius temperature as this *T_A_* can change with the life stage of a species. We therefore support the idea that two sets of Arrhenius temperatures should be used in all intertidal Lophotrochozoan species that have a larval life in pelagic area when using an abj-DEB model. As the authors are aware only one Arrhenius temperature is usually including into any DEB model even if, a species may experience different temperature ranges during their life cycle. For instance, in the mollusc bivalve *Magdallena gigas*, which is a commercial species and intertidal, in the AMP database, the value of *T_A_* is set at 8000 K despite that Rico-Villa *et al.* (2010) calculated a higher value of *T_A_* (11 000 K) for the larvae after rearing them at five different temperatures from 17°C to 32°C.

*Arenicola marina* has been cultured since the late 90s in Northeast England (Northumbland Seabait ltd.) with a number of patents issued from this bait farming [e.g. Olive *et al.*, 2001 (WO2003007701A2); Craig and Olive, 2005 (WO2005043994A1)]. The initial purpose of the culture of lugworms in the UK was to support the demands of fishermen that were digging intensively the worms used for bait (Olive, 1993, 1994; Olive and Cowin, 1994). Recently, *A. marina* is reared in a farm in Noirmoutiers Island in West of France (Hemarina Ltd™) for medical purposes, where numerous of exiting research is carried out on the medical potential and application of the lugworm haemoglobin (Asong-Fontem *et al.*, 2021; Batool *et al.*, 2021; Le Daré *et al.*, 2021; Le Meur *et al.*, 2021). Our study on larval physiology highlight that the optimal temperature for growth is ~15°C with a maximal tolerance of 21°C and this could have interesting application in aquaculture.

## Conclusion

Overall, our data on the early larval stages of *A. marina* will be valuable in improving the existing abj-DEB model for this engineer species. These include not only life traits such as age at birth and size at birth but also Arrhenius temperatures and length over time for two food levels. DEB modelling allows to predict functional traits of the species such as size at first maturity, life span, number of oocytes during the whole life cycle (total reproductive output), growth rate, maximum length (Lmax), etc. These model outputs can help marine conservation managers make decisions to preserve the *A. marina* population exploited by bait fishing. In particular, it helps stakeholders to establish regulatory measures such as catch size limits or the number of individuals that can be harvested. One of the solutions to overexploitation of lugworms is aquaculture farming. Our data underline that the optimal temperature for rearing lugworm larvae is 15°C and that it is necessary to feed them with microalgae after the ‘birth’ period, which occurs 17 days after fertilization.

## Funding

This work has been financially supported by the European Union (ERDF), the French State, the French Region Hauts-de-France and Ifremer, in the framework of the project CPER MARCO 2015–2021.

## Conflicts of interest statements

The authors declare no financial and personal conflict of interest.

## Data availability

The data underlying this article are available in the article.
